# Associations of employment status and educational levels with mortality and hospitalization in the dialysis outcomes and practice patterns study in Japan

**DOI:** 10.1371/journal.pone.0170731

**Published:** 2017-03-06

**Authors:** Yasuo Imanishi, Shingo Fukuma, Angelo Karaboyas, Bruce M. Robinson, Ronald L. Pisoni, Takanobu Nomura, Takashi Akiba, Tadao Akizawa, Kiyoshi Kurokawa, Akira Saito, Shunichi Fukuhara, Masaaki Inaba

**Affiliations:** 1 Department of Metabolism, Endocrinology and Molecular Medicine, Osaka City University Graduate School of Medicine, Osaka, Japan; 2 Department of Healthcare Epidemiology, School of Public Health in the Graduate School of Medicine, Kyoto University, Kyoto, Japan; 3 Arbor Research Collaborative for Health, Ann Arbor, MI, United States of America; 4 University of Michigan, Ann Arbor, MI, United States of America; 5 Medical Affairs, Kyowa Hakko Kirin, Co. Ltd., Tokyo, Japan; 6 Department of Blood Purification and Internal Medicine, Kidney Center, Tokyo Women's Medical University, Tokyo, Japan; 7 Division of Nephrology, Department of Medicine, Showa University School of Medicine, Tokyo, Japan; 8 National Graduate Institute for Policy Studies, Tokyo, Japan; 9 Division of Nephrology and Dialysis Center, Shonantobu General Hospital, Kanagawa, Japan; 10 Center for Innovative Research for Communities and Clinical Excellence, Fukushima Medical University, Fukushima, Japan; Tokushima University Graduate School, JAPAN

## Abstract

**Background:**

Socioeconomic status (SES) factors such as employment, educational attainment, income, and marital status can affect the health and well-being of the general population and have been associated with the prevalence of chronic kidney disease (CKD). However, no studies to date in Japan have reported on the prognosis of patients with CKD with respect to SES. This study aimed to investigate the influences of employment and education level on mortality and hospitalization among maintenance hemodialysis (HD) patients in Japan.

**Methods:**

Data on 7974 HD patients enrolled in Dialysis Outcomes and Practice Patterns Study phases 1–4 (1999–2011) in Japan were analysed. Employment status, education level, demographic data, and comorbidities were abstracted at entry into DOPPS from patient records. Mortality and hospitalization events were collected during follow-up. Patients on dialysis < 120 days at study entry were excluded from the analyses. Cox regression modelled the association between employment and both mortality and hospitalization among patients < 60 years old. The association between education and outcomes was also assessed. The association between patient characteristics and employment among patients < 60 years old was assessed using logistic regression.

**Results:**

During a median follow-up of 24.9 months (interquartile range, 18.4–32.0), 10% of patients died and 43% of patients had an inpatient hospitalization. Unemployment was associated with mortality (hazard ratio [HR] = 1.57; 95% confidence interval [CI]: 1.05–2.36) and hospitalization (HR = 1.25; 95% CI: 1.08–1.44). Compared to patients who graduated from university, patients with less than a high school (HS) education and patients who graduated HS with some college tended to have elevated mortality (HR = 1.41; 95% CI, 1.04–1.92 and HR = 1.36; 95% CI: 1.02–1.82, respectively) but were not at risk for increased hospitalizations. Factors associated with unemployment included lower level of education, older age, female gender, longer vintage, and several comorbidities.

**Conclusions:**

Employment and education status were inversely associated with mortality in patients on maintenance HD in Japan. Employment but not education was also inversely associated with hospitalizations. After adjustment for comorbidities, the associations with clinical outcomes tended to be stronger for employment than education status.

## Introduction

It has been reported that socioeconomic status (SES) factors such as employment, educational attainment, income, and marital status affect the health and well-being of the general population and public health [1] and may serve as social determinants of health [2].

SES is associated with the prevalence of chronic kidney disease (CKD) [3]. In a retrospective longitudinal study in the United Kingdom, the increase in local poverty based on deprivation scores was reportedly associated with an increase in CKD incidence [4], and in the ARIC study conducted in the United States (US), an increased risk (2.1 times) of CKD progression in Caucasian men living in low SES areas was reported in the area-level investigation of SES, which was based on the national census data [5]; thus, the association between residing in a low SES area and CKD incidence and progression has become clear. In a prospective longitudinal study in the US, the risk of CKD progression in a population with low annual income increased more than that in a population with high annual income [6]. In a population-based case-control study in Sweden, CKD was reportedly more likely to progress in less skilled workers and less educated populations than in their counterparts [7]. Thus, the importance of individual SES has been pointed out.

With regard to CKD, the social adaptability index (SAI), which is calculated by the scoring of five SES factors, i.e., employment status, education level, marital status, substance abuse, and income, has been proposed. A significant association between SAI and mortality was shown in patients with stage 2–5 CKD who are not on dialysis [8] and those with stage 5 CKD who are on dialysis [9]. However, no studies to date in Japan have reported on the prognosis of patients with CKD with respect to SES.

It became clear in the Dialysis Outcomes and Practice Patterns Study (DOPPS) that the prognoses of patients on maintenance dialysis differ among countries and are associated with differences in practice patterns [10]. Given the lack of information about SES and clinical outcomes for maintenance hemodialysis patients in Japan, we used the Japan DOPPS (J-DOPPS) to investigate associations of SES with mortality and hospitalization with particular focus on employment and education.

## Subjects and methods

### Study design

The DOPPS is an international prospective cohort study of hemodialysis (HD) patients ≥ 18 years of age. Patients in the DOPPS are enrolled randomly from a representative sample of dialysis facilities within each country [11,12]. This analysis includes data from 7974 HD patients in Japan: 2315 from 65 facilities in phase 1 (1999–2001), 1693 from 60 facilities in phase 2 (2002–2004), 2068 from 62 facilities in phase 3 (2005–2008), and 1898 from 59 facilities in phase 4 (2009–2011). DOPPS study include representative samples of Japanese dialysis patients by stratified random sampling method. Facilities are randomly selected from a national list of dialysis facilities. Study approval was obtained from a central institutional review board in Tokyo Women's Medical University (Approval Numbers 709, 1178, 1278, 1527, 1826, and 2143). Additional study approval and written patient consent were obtained as required by national and local ethics committee regulations. Employment status, education level, demographic data, and comorbid conditions were abstracted from patient records. Mortality and hospitalization events were collected during study follow-up. Patients on dialysis < 120 days at study entry were excluded from the analyses because higher mortality was observed soon after the initiation of HD [13]. Patients for whom data were missing on age or gender were also excluded (N = 7).

### Variables

The DOPPS collects information on social factors on the baseline medical questionnaire (MQ) and/or the self-administered patient questionnaire (PQ), which is typically administered within 1 month after study entry (median, 1.0 months; interquartile range [IQR], 0.6–1.6). Employment (yes/no) was defined for patients < 60 years of age using the MQ: patients working part- or full-time were classified as employed. Patients ≥ 60 years old were excluded from the employment analyses because they are not expected to be employed in Japan. Education level was categorized into three groups by the MQ: (1) less than high school graduate; (2) high school graduate or some college; and (3) university graduate. In DOPPS phases 3–4, additional categories for junior college graduate and technical college graduate were added to the questionnaire; we included these patients in category (2) because they were not university graduates. Data from the PQ administered within 1 year of baseline were used to supplement education level and employment status if missing on the MQ. Hospitalization events were defined as an inpatient hospitalization with an overnight stay; study facilities not reporting any hospitalization events were excluded from the analyses pertaining to hospitalizations because of suspected under-reporting.

### Statistical analysis

Patient characteristics including demographics and comorbidities were reported for the overall sample as well as by employment status and education level. Cox regression was used to model the association between employment and both mortality and time to first hospitalization among patients < 60 years old. The association between education and outcomes was assessed among all patients with education level data, with university graduate considered the reference group. Cox models were analysed with varying levels of covariate adjustment: (1) crude model stratified by DOPPS phase; (2) plus adjustments for age, gender, and vintage; and (3) plus adjustments for 13 summary comorbid conditions listed in Table 1. Analyses were repeated separately among men and women. Interaction effects were tested between gender and both employment and education. All Cox models accounted for facility clustering using robust sandwich covariance estimators. Time at risk started at study enrolment and ended at the time of death, 7 days after leaving the facility due to transplant or transfer, 7 days after changing modality, loss to follow-up or end of DOPPS phase. The proportional hazards assumption was tested and confirmed using log (time) interactions and visually inspecting log-log survival plots.

**Table 1:** Patient characteristics overall, and by employment and education status. Reanalyzed the data and re-evaluate the impact of employment in patients younger than age 60. The table was also replaced.

		All	Age < 60 with employment data			Patients with education data			
Patient characteristic (mean ± SD or %)		All patients	All	Not employed	**Employed**	All	University graduate	HS or some college	Less than high school
N patients		7974	3151	1534 (49%)	1617 (51%)	6413	966 (15%)	3326 (52%)	2121 (33%)
Age (years)		61.7 ± 12.7	49.8 ± 7.9	51.2 ± 7.2	48.4 ± 8.3	60.9 ± 12.6	58.3 ± 12.3	58.5 ± 12.5	65.8 ± 11.3
Gender (% male)		62%	63%	41%	83%	63%	87%	59%	57%
Vintage (years)		7.5 ± 6.9	8.7 ± 7.4	8.9 ± 7.6	8.6 ± 7.2	7.6 ± 7.0	7.3 ± 6.8	8.2 ± 7.3	7.0 ± 6.4
Smoking status (%)									
	Active smoker	23%	30%	23%	38%	23%	22%	24%	22%
	Former smoker	16%	13%	10%	15%	17%	24%	17%	14%
	Never smoker	61%	57%	67%	47%	60%	54%	59%	64%
Comorbid conditions (%)									
	Coronary artery disease	29%	21%	23%	19%	30%	27%	29%	32%
	Cancer (non-skin)	8%	5%	6%	4%	8%	9%	8%	8%
	Other cardiovascular disease	30%	21%	22%	19%	29%	29%	28%	32%
	Cerebrovascular disease	14%	8%	12%	5%	14%	12%	13%	16%
	Coronary heart failure	17%	12%	14%	10%	16%	17%	15%	19%
	Diabetes	31%	23%	29%	18%	30%	27%	28%	34%
	Gastrointestinal bleeding	5%	4%	5%	3%	4%	5%	4%	5%
	Hypertension	68%	63%	64%	62%	68%	73%	67%	69%
	Lung disease	3%	1%	1%	1%	2%	2%	2%	3%
	Neurologic disease	8%	3%	6%	2%	6%	4%	5%	9%
	Psychiatric disorder	4%	4%	6%	3%	3%	3%	4%	3%
	Peripheral vascular disease	15%	11%	14%	8%	15%	14%	14%	16%
	Recurrent cellulitis/gangrene	4%	3%	4%	2%	3%	4%	3%	3%

Mean ± standard deviation, or % shown.

The association between patient characteristics and employment status was assessed using generalized estimating equations models with logit link function and accounted for facility clustering effects. Each patient characteristic included in Table 1, plus education, was tested in a separate model with employment (yes/no) as the outcome and adjustment for DOPPS phase, age, and gender. We also tested all listed covariates in a mutually adjusted model. In this single model, education level was the only variable with missing data (637/4351; 15%) and missing values were treated as a separate category to prevent a further sample size loss; a sensitivity analysis showed that the results were similar to a complete-case analysis. All statistical analyses were performed using SAS version 9.4 (SAS Institute, Cary, NC, USA).

## Results

### Descriptive analyses

The study sample for analysis included 7974 patients. Among the 3151 patients < 60 years for whom employment data were available, 44% were employed at study entry. Among 6413 patients for whom education data were available, 15% were university graduates, 52% were high school graduates and/or had some college experience, and 33% had less than a high school education. Table 1 shows the overall patient characteristics by employment status and education level. Even when restricted to patients < 60 years, those who were employed tended to be much younger (mean, 48.4 vs. 51.2 years) than those unemployed. Patients who were employed were also much more likely to be men: 83% in the employed group vs. 41% in the unemployed group. Many of the summary comorbid conditions tended to be more prevalent among unemployed patients, including diabetes, cerebrovascular disease, and peripheral vascular disease. Patients with a university degree had the same mean age as those with a high school degree and/or some college (mean, 58.3 vs. 58.5 years), but both groups tended to be much younger than those without a high school degree (mean, 65.8 years). University graduates were predominantly male (87%), while the other education groups were 55–60% male. The prevalence of some comorbid conditions tended to be higher among patients with less education, but these crude associations may be confounded by age.

### Associations with mortality and time to first hospitalization

Median follow-up during the study was 24.9 months (IQR, 18.4–32.0); during this time, 794 patients died (10%). Nine facilities did not report any hospitalization events in a particular study phase; among the 7726 patients for whom hospitalization data were available, 3359 (43%) had an inpatient hospitalization recorded. The associations of both employment and education with mortality are described in Table 2. In a crude model, patients who were unemployed had a higher risk of mortality (hazard ratio [HR], 1.88; 95% confidence interval [CI], 1.32–2.68). After adjustment for basic demographics, the HR remained 2.13. After adjustment for comorbidities, the HR was attenuated to 1.57 (95% CI, 1.05–2.36). Education less than high school, compared to university graduate, was strongly associated with mortality in a crude model (HR, 1.98; 95% CI, 1.46–2.69). This association was attenuated by the adjustment for demographic factors, primarily age. In the fully adjusted model considering university graduate as the reference group, education less than high school (HR, 1.41, 95% CI, 1.04–1.92) and high school graduate and/or some college (HR, 1.36; 95% CI, 1.02–1.82) were associated with increased mortality.

**Table 2:** Association between social factors and mortality (hazards ratio), by level of adjustment. Reanalyzed the data and re-evaluate the impact of employment in patients younger than age 60. The table was also replaced.

		Crude	+ adjusted for age,gender, vintage	+ adjusted for 13 comorbidities
**Employment (age < 60)**				
*N = 3151; N = 132 events (4%)*				
	Employed	1 (Ref.)	1 (Ref.)	1 (Ref.)
	Not employed	1.88 (1.32–2.68)	2.13 (1.45–3.14)	1.57 (1.05–2.36)
**Education**				
*N = 6413; N = 544 events (8%)*				
	Less than high school	1.98 (1.46–2.69)	1.42 (1.05–1.93)	1.41 (1.04–1.92)
	HS or some college	1.32 (0.98–1.77)	1.37 (1.02–1.84)	1.36 (1.02–1.82)
	University graduate	1 (Ref.)	1 (Ref.)	1 (Ref.)

HR (95% CI) of mortality shown; comorbidities listed in Table 1.

All models stratified by DOPPS phase and accounted for facility clustering.

Analyses of both employment and education with time to first hospitalization are described in Table 3. In an adjusted model, unemployed patients were at higher risk of hospitalization than employed patients (HR, 1.25; 95% CI, 1.08–1.44). Education level had a minimal association with hospitalization: compared to university graduates, the HR for education less than high school was 1.05 (95% CI, 0.92–1.21), while the HR for high school graduate and/or some college completed was 1.03 (95% CI, 0.92–1.17).

**Table 3:** Association between social factors and first hospitalization, by level of adjustment. Reanalyzed the data and re-evaluate the impact of employment in patients younger than age 60. The table was also replaced.

		Crude	+ adjusted for age,gender, vintage	+ adjusted for 13 comorbidities
**Employment (age < 60)**				
*N = 3035; N = 1073 events (35%)*				
	Employed	1 (Ref.)	1 (Ref.)	1 (Ref.)
	Not employed	1.37 (1.22–1.55)	1.40 (1.21–1.62)	1.25 (1.08–1.44)
**Education**				
*N = 6223; N = 2719 events (44%)*				
	Less than high school	1.28 (1.12–1.46)	1.10 (0.95–1.26)	1.05 (0.92–1.21)
	HS or some college	1.06 (0.94–1.20)	1.06 (0.94–1.19)	1.03 (0.92–1.17)
	University graduate	1 (Ref.)	1 (Ref.)	1 (Ref.)

HR (95% CI) of time to first hospitalization shown; comorbidities listed in Table 1.

All models stratified by DOPPS phase and accounted for facility clustering.

Table 4 shows the adjusted analyses of both mortality and time to first hospitalization by gender. We did not detect an interaction effect between gender and employment for either mortality (p = 0.7) or hospitalization (p = 0.3). The association between lower education level and mortality appeared to be stronger among men, but we were unable to detect an interaction effect (p = 0.4); a lack of variation, i.e., 87% of university graduates were men, likely inhibited our ability to sufficiently power a statistical test of the interaction.

**Table 4:** Association between social factors and clinical outcomes, by gender. Reanalyzed the data and re-evaluate the impact of employment in patients younger than age 60. The table was also replaced.

		All-cause mortality		Time to first hospitalization	
		Male	Female	Male	Female
**Employment (age < 60)**					
*N patients (% event)*		*N = 1976 (5%)*	*N = 1175 (4%)*	*N = 1899 (36%)*	*N = 1136 (35%)*
	Employed	1 (Ref.)	1 (Ref.)	1 (Ref.)	1 (Ref.)
	Not employed	1.49 (0.94–2.36)	1.94 (0.65–5.77)	1.29 (1.08–1.53)	1.21 (0.93–1.59)
**Education**					
*N patients (% event)*		*N = 4012 (9%)*	*N = 2401 (8%)*	*N = 3896 (44%)*	*N = 2327 (44%)*
	Less than high school	1.42 (1.00–2.00)	1.14 (0.50–2.57)	1.03 (0.89–1.20)	1.21 (0.88–1.66)
	HS or some college	1.48 (1.08–2.03)	0.98 (0.42–2.26)	1.03 (0.90–1.17)	1.15 (0.84–1.57)
	University graduate	1 (Ref.)	1 (Ref.)	1 (Ref.)	1 (Ref.)

HR (95% CI) of mortality or time to first hospitalization shown.

All models stratified by DOPPS phase and accounted for facility clustering.

Models adjusted for demographics (age, vintage) and 13 comorbidities listed in Table 1.

### Patient characteristics associated with employment

Modeling unemployed vs employed as a binary outcome, we used a set of minimally adjusted models, adjusted only for DOPPS phase, age, and gender, as well as a single fully adjusted model to characterize variables associated with employment among patients < 60 years old (Table 5). Of all tested variables, gender had the strongest association by far with employment; the odds ratio (OR) of not being employed was 0.11 (95% CI, 0.09–0.14), i.e., men had about an eight times higher odds of being employed as women. Age and education were also strongly associated with employment; the OR of not being employed was 1.49 (95% CI, 1.32–1.67) per 10 years older, and compared to the reference group of university graduates, the OR was 5.21 (95% CI, 3.77–1.19) for less than high school and 2.72 (95% CI, 2.07–3.57) for high school graduate and/or some college completed. Among the 13 comorbidities tested in the adjusted model, neurological disease (OR, 3.02; 95% CI, 1.71–5.34), cerebrovascular disease (OR, 2.39; 95% CI, 1.72–3.34), diabetes (OR, 2.16; 95% CI, 1.71–2.75), psychiatric disorder (OR, 1.96; 95% CI, 1.19–3.23), and peripheral vascular disease (OR, 1.53; 95% CI, 1.15–2.03) were most strongly associated with unemployment.

**Table 5:** Association between patient characteristics and employment among patients < 60 years old. Reanalyzed the data and re-evaluate the impact of employment in patients younger than age 60. The table was also replaced.

Patient characteristic		Crude models	Adjusted model
Age (per 10 years)		1.70 (1.53,1.89)	1.49 (1.32,1.67)
Male (vs. female)		0.13 (0.11,0.16)	0.11 (0.09,0.14)
Vintage (per 5 years)		1.00 (0.94,1.06)	1.08 (1.01,1.15)
Comorbid conditions			
	Coronary artery disease	1.34 (1.09,1.65)	1.01 (0.81,1.27)
	Cancer (non-skin)	1.00 (0.68,1.46)	0.87 (0.60,1.28)
	Other cardiovascular disease	1.24 (1.01,1.51)	1.04 (0.84,1.29)
	Cerebrovascular disease	3.12 (2.27,4.29)	2.39 (1.72,3.34)
	Coronary heart failure	1.59 (1.22,2.07)	1.19 (0.88,1.60)
	Diabetes	2.49 (2.05,3.03)	2.16 (1.71,2.75)
	Gastrointestinal bleeding	1.91 (1.15,3.16)	1.62 (0.98,2.67)
	Hypertension	1.17 (0.98,1.39)	1.04 (0.86,1.27)
	Lung disease	1.30 (0.62,2.72)	0.86 (0.38,1.91)
	Neurologic disease	4.67 (2.73,8.00)	3.02 (1.71,5.34)
	Psychiatric disorder	2.51 (1.58,3.99)	1.96 (1.19,3.23)
	Peripheral vascular disease	2.32 (1.80,2.99)	1.53 (1.15,2.03)
	Recurrent cellulitis/gangrene	2.36 (1.44,3.85)	1.10 (0.63,1.93)
Education			
	Less than high school	5.57 (4.09,7.59)	5.21 (3.77,7.19)
	HS or some college	2.65 (2.04,3.43)	2.72 (2.07,3.57)
	University graduate	1 (Ref.)	1 (Ref.)

OR (95% CI) of unemployment; model restricted to patients < 60 years old (N = 3135).

Crude models are only adjusted for DOPPS phase, gender, age.

Adjusted model is simultaneously adjusted for all variables in the table.

## Discussion

Both employment and education status were associated with mortality; however, with regard to hospitalization, employment status and not education was a risk factor among maintenance HD patients in Japan. Factors associated with unemployment included lower education, older age, female gender, longer vintage, and several comorbid conditions.

Since employment and education may be viewed as markers of income, it is relevant that an association between income inequality and life expectancy has been reported [14]. Lower levels of income inequality are associated with better child well-being in the 2013 UNICEF Index of Child Well-being in 21 wealthy countries; Japan is among the best countries in terms of child well-being and has relatively low income inequality [15].

However, even in Japan, where income differences are small, the SES factors, such as employment, educational attainment, and income are associated with various health parameters, such as mortality [16]. With regard to patients on maintenance dialysis, comparison of health-related quality of life (QOL) among Japan, the US, and Europe also showed that dialysis patients in Japan have significantly higher income and employment levels [17] and a more favourable prognosis [18] than their counterparts in the US and Europe. However, the present study showed that, even in Japan where the SES is high, employment and educational statuses among people belonging to other SESs were associated with prognosis. Because we analysed only Japan data in this study, we can’t know any difference between Japan and other countries. In the future study, we would like to examine those associations using international data.

Even after the adjustment for demographics and co-morbidities, strong associations between unemployment and both all-cause mortality and hospitalization were observed; an association between education and all-cause mortality was also observed (Tables 2 and 3). For employment, adjustment for demographics did not change the all-cause mortality HR; perhaps because employment was associated with both younger age (lower mortality) and male gender (higher mortality), so that the confounding impacts by these two variables cancelled out. A large change in the magnitude of the HR was observed after adjustment for comorbidities, which were mostly inversely associated with employment and positively associated with mortality. These results suggest that comorbidities can be confounding factors which affect both employment status and clinical outcomes. On the other hand, the all-cause mortality HR for less than HS education was mostly attenuated by adjustment for demographics, likely explained by the 7.5 year age difference between groups (Table 1); further adjustment for comorbidities had minimal impact. Thus, the mechanism of association between individual SES and prognosis seems to differ. The association between lower education level and mortality appeared to be stronger among men, but we were unable to detect an interaction effect due to sparse cells. In this sample, a much higher percentage of men are university graduates (21% vs. 5%); this issue of statistical power is compounded by fewer adverse events occurring among women. Thus, we cannot conclude whether there were any differences in the associations between SES and clinical outcomes between men and women.

With regard to employment and income, which is considered important because of its association with prognosis, an observational study in chronic dialysis patients was conducted in Argentina, where public health insurance coverage is insufficient. This study reported an association between no income and high all-cause mortality [19]. A significant association between income and mortality is also reported in patients with stage 3–4 CKD [20]. In Japan, where most of the medical cost of maintenance dialysis is covered by public health insurance, the possibility that the medical care would deteriorate with decreasing income is low. However, the possibility that low income due to unemployment may have suppressed other health-related expenditures and, thus, causing poor prognosis cannot be ruled out.

The SAI is calculated by the scoring of five SES factors, i.e., employment status, education level, marital status, substance abuse, and income [8], but the ratio of substance abuse except smoking and drinking were very low in Japan. In addition, the data of substance abuse except smoking were not collected in this study. We also had many missing data on income and marital status. So only employment status and education level was employed in this study.

By examining the factors associated with unemployment, aging, long-term dialysis, gender, and various comorbidities (cerebrovascular disease, diabetes, neurological disease, psychiatric disorder, and peripheral vascular disease) were found to be the physical factors that impede employment (Table 5). Especially, diabetic patients with complications such as diabetic retinopathy and neuropathy, may easily lose their jobs. However, because the present study investigated only comorbidities, it is impossible to examine whether job loss was due to disease development. We observed a strong association between low-level of education and unemployment (Table 5), however, this may reflect a lack of education due to insufficient educational opportunities or gender differences in educational opportunities.

Our finding of a significant association between education and all-cause mortality agrees with findings in the general population in Japan [21,22]. Outside Japan, an association with decreased access to health care is reportedly a mechanism of poor prognosis due to poor education [2]. However, because the number of visits by maintenance dialysis patients is three per week, it is difficult to consider that the frequency of access to health care is affected by income. Therefore, factors other than access to health care, such as health literacy, may be involved. Educational level is reportedly associated with communication ability and health literacy, which are considered important factors of access to health care [23] and health communication outcomes [24].

The associations between low SES and bad health-related behaviours, such as poor dietary habits [25,26,27],physical inactivity, and smoking, have been studied in the general population [28], suggesting that these unhealthy behaviours may also be affecting maintenance dialysis patients.

The dietary habits in hemodialysis patients were examined in the DOPPS and other studies [29,30,31], and documented associations of all-cause mortality and various nutrient indices (serum creatinine, serum albumin, normalized protein catabolic rate, body mass index, and cachexia) [30] and also with anorexia [31]. Thus, nutrient intake and nutritional status are important prognosis-related factors in maintenance dialysis patients.

Summary, we examined employment status and education level as SES factors in Japanese patients on maintenance dialysis, and showed that these factors are strongly associated with all-cause mortality, and in the case of employment, also with hospitalization. The effect of employment status appears to be stronger than that of education level. The involvement of income in employment and the involvement of health literacy may be potential factors affecting outcomes; however, further studies are warranted. Although it is impossible to improve employment and education in a dialysis patient, improvements in patient prognosis may be possible by recognizing the potential role of these SES factors and possibly related patients’ health-related behaviours.
